# A Case Report of Encephalopathy With Myoclonus: A Rare Neurologic Side Effect of Ranolazine

**DOI:** 10.7759/cureus.79503

**Published:** 2025-02-23

**Authors:** Shaheer Arif, London Spears, Kenneth Shauger, Abdul Munim

**Affiliations:** 1 Department of Neurology, University of Tennessee Health Science Center (UTHSC), Memphis, USA; 2 Department of Psychiatry, University of Tennessee Health Science Center (UTHSC), Memphis, USA; 3 Department of Neurology, Veterans Affairs Medical Center, Memphis, USA; 4 Department of Medicine, Pakistan Air Force (PAF) Hospital, Islamabad, PAK

**Keywords:** clinical case report, drug adverse effect, encephalopathy, myoclonus, ranolazine

## Abstract

The evaluation of the cause of an acute encephalopathy can be challenging due to nonspecific presentations and many potential etiologies. Ranolazine-induced encephalopathy has seldom been reported in the literature. We report a case of ranolazine-induced encephalopathy with myoclonus.

A 78-year-old male with past medical history of coronary artery disease (CAD) with refractory angina on ranolazine, chronic kidney disease (CKD) stage III, multiple other medical comorbidities presented to the hospital after a fall and complaints of generalized weakness. The patient, during admission, developed encephalopathy and generalized myoclonus that resolved by stopping ranolazine.

Acute encephalopathy has a wide differential diagnosis. The association of myoclonus and bilateral asterixis favors a systemic metabolic process or a circulating factor. It is essential in workup that close attention be paid to medication review as patients with liver and/or kidney dysfunction can become toxic on routine medication doses. This case signifies the importance of medication review and highlights ranolazine as a potential cause of acute encephalopathy with myoclonus.

## Introduction

Encephalopathy is a disturbance in brain function that causes changes in behavior, level of alertness, attention, cognition, and memory and can range from mild confusion to deep coma. The pathologic process typically affects the brain globally, though more regional, focal, or multifocal lesions may be implicated. Depending upon the pace of onset and duration, it can be subclassified into hyperacute, acute, subacute, and chronic. The etiology can be divided into primary brain insult or systemic cause [1].

Acute encephalopathy refers to an alteration in mental status that occurs over hours to days. The primary neurological reasons for this can be grouped into vascular (e.g., subdural hematoma), infectious (meningitis and/or encephalitis), and inflammatory (demyelinating condition, autoimmune etiologies). Systemic include systemic infection, autoimmune diseases, acute renal or hepatic failure, hyperammonemia, thyroid abnormalities, thiamine deficiency, drug or medication-related including withdrawal and overdose, toxin ingestion, and other causes [1].

Encephalopathy associated with multifocal myoclonus narrows down the differential to renal or hepatic insufficiency or drug toxicity [1-3]. A thorough medication review is always essential, given the likely culprit might not be obvious. We present a case of ranolazine-induced encephalopathy and myoclonus to raise awareness and improve outcomes of this seemingly rare entity.

## Case presentation

A 78-year-old male with a past medical history of coronary artery disease (CAD) with coronary artery bypass grafting (CABG) and percutaneous stenting, heart failure with reduced ejection fraction (HFrEF) with ejection fraction (EF) of 35-40%, atrial fibrillation, hypertension, chronic kidney disease (CKD) stage III, and opioid use disorder treated with monthly buprenorphine depot shots presented to the ER after a fall.

He had recently been admitted for generalized weakness just two weeks prior, where he was found to have an acute kidney injury (AKI) with a serum creatinine of 2.91 mg/dl (baseline ~1.6 mg/dl). At that time, it was found he had doubled his furosemide dosing and was successfully treated with intravenous hydration.

**Figure 1:**
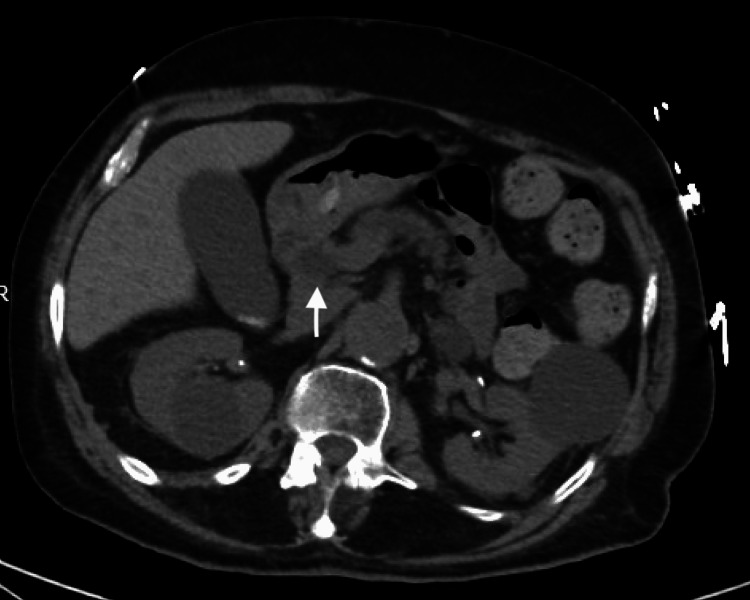
CT Abdomen without contrast showing dilated pancreatic duct (white arrow)

On his return to the ER, he stated his weakness progressed since being discharged and resulted in a fall from which he couldn't stand back up. In addition, he complained of increasing dyspnea on exertion and diffuse abdominal pain for the prior four days. Admission labs were significant for AKI, with a serum creatinine of 1.96 mg/dl, a lipase of 23 U/L, an elevated brain natriuretic peptide (BNP) of 871 pg/ml, and troponin of 44.6 ng/L noted to be down trending. He was admitted to the medicine floor with a plan for magnetic resonance cholangiopancreatography (MRCP) to evaluate his abdominal pain, given the prominence of his pancreatic and extrahepatic ducts noted on CT abdomen (Figure 1).

On the second night after admission, the patient was noted to be agitated and confused by the nursing staff and was provided a 1:1 sitter. On examination by the primary team the next morning, the patient was noted to be somnolent but was able to be aroused and followed commands. The myoclonic activity was seen, which prompted a neurology consultation (Video 1).

**Video 1:** Ranolazine induced multifocal myoclonus in setting of AKI on CKD AKI - acute kidney injury; CKD - chronic kidney disease

On initial neurological examination, he was found to be somnolent but arousable. He followed simple motor commands bilaterally. The patient had bilateral asterixis. Asynchronous, intermittent jerking was noted predominantly in the upper extremities, though activity was also seen in the lower extremities and was more notable when the patient was asleep. By this time, the patient had received a 250cc IV fluid bolus for his AKI after his creatinine had risen to 2.08 mg/dl a day after admission. On review of his medication list for probable causes of acute encephalopathy, ranolazine stood out as a possible causative agent. He was taking 1500 mg twice a day of ranolazine. Ranolazine was held on the day of consultation.

On return to see the patient the following morning, his mentation had markedly improved, and the severity of his myoclonus and asterixis were significantly reduced. He was no longer somnolent and was consistently alert and oriented throughout the interview. His AKI had resolved with a serum creatinine of 1.46 mg/dl. Ammonia was 16.6 µmol/L, and his EEG showed no evidence of cortical myoclonus or seizure. Two days after stopping ranolazine, the patient had returned to baseline completely, with no myoclonus nor asterixis noted on the exam.

Ranolazine toxicity in the setting of AKI was determined as the cause of his acute encephalopathy with myoclonus, though a definitive conclusion was limited due to not having documented ranolazine levels in the toxic range. We considered the possible contribution of narcotics but felt this was not likely. He had not filled a benzodiazepine prescription for several months and had only missed his buprenorphine depot shot by a few days due to being an inpatient at the time of his appointment. His abdominal pain resolved and workup for it was unremarkable. The patient was discharged home with home health, and ranolazine was not restarted on discharge.

## Discussion

Drugs, both prescription and illicit, are responsible for a significant burden of encephalopathy in hospitalized patients. The risk of neurotoxicity from medications increases in the elderly population with an increasing number of comorbidities, widespread polypharmacy, decreased renal clearance, and high prevalence of dementia (a major risk factor for delirium) [4].

Chronic kidney disease, in many cases, causes decreased clearance and, therefore, higher serum drug concentrations, leading to the neurotoxicity of medications that would normally be well-tolerated. All these things need to be taken into consideration when evaluating the delirious/encephalopathic patient.

Different classes of medications can lead to encephalopathy, including anti-epileptic medications, antibiotics, benzodiazepines, narcotics, antihistamines, anticholinergics, and antipsychotics amongst the better known [1,5,6].

If myoclonus is present in addition to encephalopathy, this might help confine the potential culprit. The classes of drugs that have been associated with myoclonus include opioids, antidepressants (selective serotonin reuptake inhibitors, tricyclic antidepressants, lithium), antipsychotics, antibiotics (b-lactam and quinolones), and N-methyl-D-aspartate (NMDA) receptor antagonists (memantine and amantadine) [7].

Ranolazine is an anti-anginal drug. It acts through a multitude of mechanisms, one of which is sodium (Na) channel blockade. It is thought to lead to early inactivation of Na channels and prevent their transition to a resting state [8]. Resurgent Na currents have been implicated in various Na channel mutations that cause pathology [9]. It is known that NaV 1.1 channel mutation causes severe myoclonic epilepsy in infancy. The exact mechanism of how ranolazine causes central nervous system toxicity (CNS), including myoclonus, though not known, might have to do with resurgent Na currents causing excess neuronal excitability and repetitive neuronal firing.

Metabolism of ranolazine occurs primarily through cytochrome P450 (CYP) 3A family enzymes within the liver and five to ten percent is excreted unchanged by the kidneys [10]. The drug tends to accumulate in kidney dysfunction [10]. Ranolazine is typically started at 500mg twice a day and can be increased to 1000mg twice a day. Our patient was on the highest dose of 1500 mg twice a day, and we believe CKD was a major contributing factor to the neurologic symptoms.

Symptoms of ranolazine CNS toxicity include hallucinations, tremors, dysarthria, and dysmetria [11,12]. It has seldom been documented to cause myoclonus. We found one documented case in the literature [13]. Our case adds to the prior report and highlights ranolazine as one potential cause of drug-induced myoclonus.

## Conclusions

Acute encephalopathy can have a myriad number of causes. During workup, a thorough medication review is essential in the assessment of patients. Ranolazine is an uncommon cause of encephalopathy with multifocal myoclonus, which should be considered, especially in the setting of kidney dysfunction, and efforts to increase awareness of this etiology amongst clinicians should be undertaken.
